# On the Limits of Alpine Plants: A Systematic Review of the Factors Behind Species' Elevational Range Limits

**DOI:** 10.1002/ece3.73183

**Published:** 2026-03-04

**Authors:** Sophie E. Weides, John‐Arvid Grytnes, Stefan Dullinger, Jonathan Lenoir, L. Camila Pacheco‐Riaño, John R. Pannell, Sonja Wipf, Sabine B. Rumpf

**Affiliations:** ^1^ Department of Environmental Sciences University of Basel Basel Switzerland; ^2^ Norwegian University of Life Sciences Ås Norway; ^3^ Department of Botany and Biodiversity Research University of Vienna Vienna Austria; ^4^ UMR CNRS 7058 ‘Ecologie et Dynamique des Systèmes Anthropisés’ (EDYSAN) Université de Picardie Jules Verne Amiens France; ^5^ Department of Biological and Environmental Sciences University of Gothenburg Gothenburg Sweden; ^6^ Gothenburg Global Biodiversity Centre Gothenburg Sweden; ^7^ Department of Ecology and Evolution University of Lausanne Lausanne Switzerland; ^8^ WSL Institute for Snow and Avalanche Research Davos Switzerland

**Keywords:** climate change, drivers of range limits, high‐elevation plants, literature review, range edges, range margins

## Abstract

Understanding the factors behind species' range limits is a fundamental objective in ecology. Recent research in alpine plant ecology has moved beyond the classical view that distributions are chiefly shaped by climate and competition. Specifically, broader sets of factors have been taken into account, comprising both biotic factors such as facilitation and herbivory as well as additional abiotic factors such as soil properties. However, an overview of the factors that have been identified and studied as important for elevational range limits of alpine plant species is lacking. In this systematic literature review, we synthesize evidence derived from 107 empirical studies on 226 vascular plant species occurring beyond elevational and latitudinal treelines. We find a persistent research focus on the upper elevational range limit (73% of the studies) and on the role of abiotic factors (54% of the studies), particularly temperature (36% of the studies), whereas research on inter‐ and intraspecific factors (40% and 25%, respectively), such as herbivory or phenology, remained comparatively rare. While temperature was clearly identified as a major factor influencing the upper range limit (29% of the studies), water availability (15% of the studies) was most commonly studied at the lower range limit. Even though a broad set of factors has been investigated, many potentially important factors remain poorly researched, such as the influence of gene flow and connectivity between populations, phenology and light (each only one or two studies). Our findings highlight the need to move beyond temperature and plant–plant interactions as factors influencing the elevational range limits of alpine plants and to integrate intraspecific (such as gene flow and adaptations) and edaphic factors more fully into future research. Improved methodological standardization and transparency and increased attention on lower range limits will be essential for explaining and predicting alpine plant responses under accelerating environmental change.

## Graphical abstract

This systematic review of 107 studies on the factors behind the elevational range limits of alpine vascular plants shows a persistent emphasis on upper limits and abiotic factors, especially temperature, while work at lower limits is more evenly distributed across water availability, plant–plant interactions, and selection/local adaptation. Inter‐ and intraspecific factors remain underrepresented, with major gaps in plant–microbe interactions, phenology and light—underscoring the need to move beyond temperature, with improved methodological specificity, and to devote more attention to lower limits.

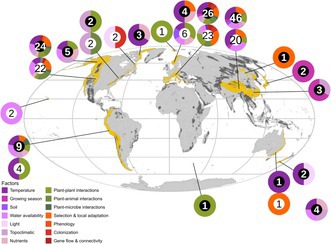

## 1 Introduction

One of the fundamental goals of ecology and biogeography is to understand the distribution of species (von Humboldt and Bonpland 1805). Historically, climate has been considered as an important factor in determining biogeographic patterns (Darwin 1859). Yet, Darwin already noted that species' distributions cannot be solely attributed to climate, and Dobzhansky (1950) and MacArthur (1972) highlighted that non‐climatic factors—particularly biotic interactions—can play a dominant role, especially at the lower edges of species' ranges. Despite extensive research and reviews on what determines species range limits over the past decades (e.g., effect of species interactions along abiotic stress‐gradients; Louthan et al. 2015), broad reviews covering multiple ecosystems (Normand et al. 2009) and taxonomic groups (Cahill et al. 2014; Louthan et al. 2015; Paquette and Hargreaves 2021), an overview of studies specific to the elevational range limits of alpine plants is lacking. This gap matters because general knowledge about species range limits cannot be directly transferred to alpine ecosystems, which are characterized by exceptionally steep and extreme gradients. Darwin (1859) acknowledged this peculiarity early on, noting that on “snow‐capped summits, […] the struggle for life is almost exclusively with the elements” (Darwin 1859, 69). Moreover, mountain ecosystems occur on all continents and across all latitudes, and even though they cover only 12% of the terrestrial land mass outside Antarctica (Körner 2021), they are biodiversity hotspots (Myers et al. 2000) and harbor disproportionately high levels of endemism (Hamid et al. 2020; Körner 2021).

For several alpine and arctic plant species, key factors that influence growth and overall performance, particularly temperature and water availability, are already comparatively well understood (Weijers et al. 2010, 2017; Liang et al. 2012; Rayback et al. 2012; Buchwal et al. 2020). At the same time, patterns of alpine plant diversity and endemism have been linked to climatic and topographic variables (Irl et al. 2015), reflecting that species differ in their sensitivity to these factors. Even though prevailing assumptions exist about what determines the elevational range limits of alpine plants, especially the upper limit, robust tests of these assumptions are scarce (Körner 2021). Regarding the upper range limit, studies have focused on abiotic factors, especially low temperature, as well as on facilitation as a biotic mitigator of abiotic stress (Wied and Galen 1998; Arroyo et al. 2003; Raath‐Krüger et al. 2019; von Büren and Hiltbrunner 2022). In this context the shrubline (i.e., the upper limit for shrubs; Huang et al. 2018), and the grassline (i.e., the upper limit of closed grasslands; Huang et al. 2018; Bürli et al. 2021), have received some attention as well. For the lower range limit, biotic interactions are considered an important factor, as alpine plants can persist in warmer temperatures when competitors are absent, for example, in botanical gardens (Vetaas 2002). In addition to these factors, water availability, particularly in the form of precipitation, has been investigated, especially in water‐limited alpine environments (Giménez‐Benavides et al. 2008; Berio Fortini et al. 2022).

Mountain ecosystems are increasingly recognized as climate‐sensitive regions that are warming faster than the global average (Harsch et al. 2009; Rahbek et al. 2019; Pepin et al. 2022; Kotlarski et al. 2023). Upslope range shifts of alpine plant species observed over the last decades can at least partially be attributed to climate change (Chen et al. 2011; Freeman et al. 2018; Rumpf et al. 2019; Lenoir et al. 2024). However, the considerable variation in both magnitude and direction of the observed range shifts (Rubenstein et al. 2023; Lawlor et al. 2024) suggests that climate warming alone cannot explain these shifts, indicating that it is not the full explanation for the position of the range limits themselves. Recent studies have therefore expanded the focus to include other abiotic (e.g., precipitation, soil characteristics, solar radiation; Normand et al. 2009; Chen, Shen, and Chan 2025), interspecific (e.g., microbial associations that buffer abiotic stress by promoting adaptation; Hou et al. 2024) and intraspecific factors (e.g., demographic constraints, dispersal limitations; HilleRisLambers et al. 2013; Louthan et al. 2015; Paquette and Hargreaves 2021; Helm et al. 2024) for both upper and lower range limits (Normand et al. 2009; Cahill et al. 2014; Louthan et al. 2015; Paquette and Hargreaves 2021). In parallel, disturbance regimes can strongly mediate climate change (Hickman et al. 2024), such as fire (in combination with herbivory; Irl et al. 2012), land‐use change (Marzini et al. 2025) or permafrost thaw (Myers‐Smith et al. 2011). Other processes that may reshape range limits include, eutrophication through nitrogen deposition (Bowman et al. 2015), mismatches between herbivore and plant responses to environmental change (Santos et al. 2015), asynchronous shifts in the phenological timing of plants and pathogens (Penczykowski et al. 2017), and the decoupling of plant–pollinator interactions (Williams et al. 2015; Kerner et al. 2023). Introductions of invasive species can further alter vegetation dynamics and change community responses to climate warming (e.g., competition with non‐native plant species (Dainese et al. 2017) or herbivory by invasive rabbits; Cubas et al. 2018; Martín‐Esquivel et al. 2020). However, no systematic literature review has been conducted on this topic to date.

Here, we undertake a systematic review of the literature on elevational range limits. Our review summarizes the available studies on the factors that control the position of upper and lower elevational range limits of alpine plants derived from observational, experimental, and modeling studies. We specifically ask: (i) what general factors have been studied to understand upper and lower elevational range limits of alpine plant species; and (ii) for each factor, which aspects were addressed and to which degree of detail the studies reported them. Our overarching objective is to identify the factors that have attracted most attention in studies of elevational range limits and to highlight factors that merit more research attention in future.

## 2 Material and Methods

**FIGURE 1:**
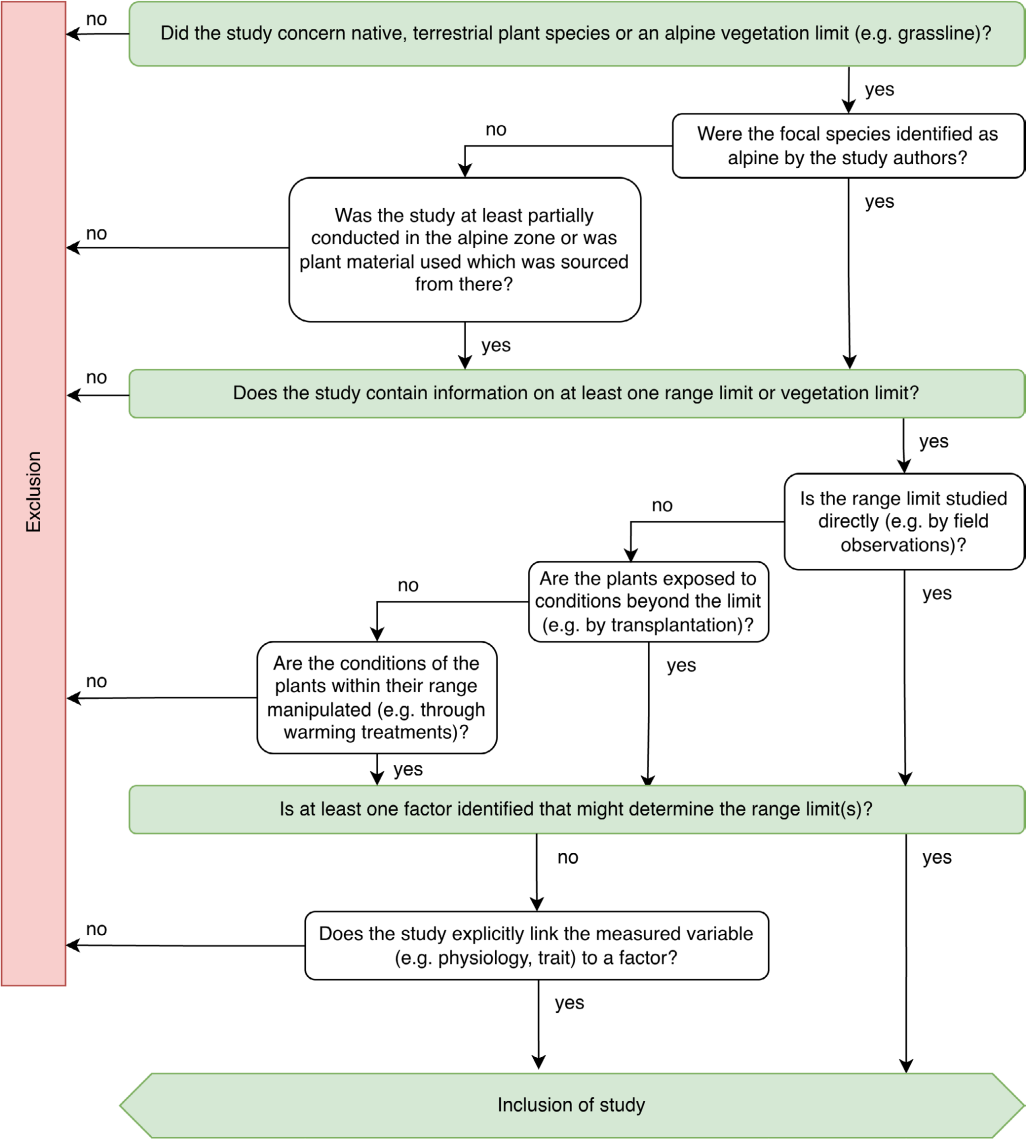
Criteria for inclusion of studies into the literature collection for assessment.

On January 15, 2026, we searched the Web of Science and Scopus for studies published in English on the elevational range limits of alpine plant species. We used a Boolean keyword combination targeting elevation‐related terms, plant‐related traits, and range limit concepts on abstracts and titles: (elevat* OR altitud* OR alpin* OR montan* OR nival* OR mountain*) AND (distribut* OR range* OR niche* OR gradient* OR shift* OR occurrence* OR abundance* OR location* OR adaptation* expan* OR retract* OR grow* OR surv* OR reproducti* OR germinati* OR speciati* OR evoluti*) AND (limit* OR edge* OR margin* OR boundar* OR border* OR constraint* OR restrain* OR restrict* OR confin* OR extrem* OR verge* OR brink* OR threshold*) AND (plant* OR vegetat* OR shrub* OR grass* OR sedge* OR herb* OR forb* OR graminoid* OR cushion*; for exact queries see Appendix A.1). This query resulted in 36,287 studies. We followed the PRISMA workflow (Page et al. 2021) to ensure scientific standards and reproducibility. First, we excluded 9127 duplicated studies and then applied a first‐pass screening of titles, abstracts and meta‐data in R to remove records clearly outside the scope of this review (4989 studies excluded; for details see Appendix A.2, Supporting Information 2). This left in total 22,171 studies that were manually evaluated for fit in this review based on pre‐defined criteria (Figure 1). To ensure the quality of the included studies, we additionally excluded studies from journals that are considered predatory (for details see Appendix A.2).

We first screened all records at the title level and excluded studies for which the title clearly indicated that the inclusion criteria were not met (17,725 studies excluded; e.g., studies on agriculture, mechanics, medicine). The remaining records were subsequently assessed based on information provided in the abstracts (3894 studies excluded). Finally, all remaining 445 studies were evaluated based on the information provided in the main text. Only 107 studies fulfilled all criteria (Figure 1) and were retained for further analysis (see Supporting Information S1).

For each study, we extracted: (i) bibliographic and methodological metadata, including author names, study year, location, number of focal species, methodological approach(es) (for details, see S5), and whether upper and/or lower range limit(s) were addressed; (ii) species identity, growth form and the life stage investigated; and (iii) factor‐specific information, including the identity, effect, and any interactions of the factors (see Appendix A: Table A1). Each combination of species, factor and study was treated as a separate record to account for within‐study heterogeneity. To illustrate the main patterns, factors were categorized into three types for subsequent analysis: abiotic (e.g., temperature, water availability), interspecific (e.g., plant–plant interactions, gene flow via hybridization/introgression), and intraspecific (e.g., selection and local adaptation, gene flow via conspecific populations, phenology).

All data processing was performed using the package tidyverse (Wickham et al. 2019), and visualizations were generated using ggplot2 (Wickham 2016) in R version 4.4.3 (R Core Team 2025).

## 3 Results

The 107 studies included in this review spanned 39 years until the beginning of 2026 and collectively reported 605 observations (i.e., unique combinations of species, factor and study), covering 266 distinct alpine plant species and two genera not specified at the species level by the study's authors. The majority of species (88%) were only investigated in one single study, but some species were investigated by multiple studies (up to a maximum of six different studies in the case of *Poa alpina* and *Ranunculus glacialis* ; for more details see Appendix B: Table A2) and on average three distinct alpine species were addressed per study. Only nine species occurred in more than two studies, and none was assessed more than four times in relation to a specific factor (Appendix B: Table A3).

**FIGURE 2:**
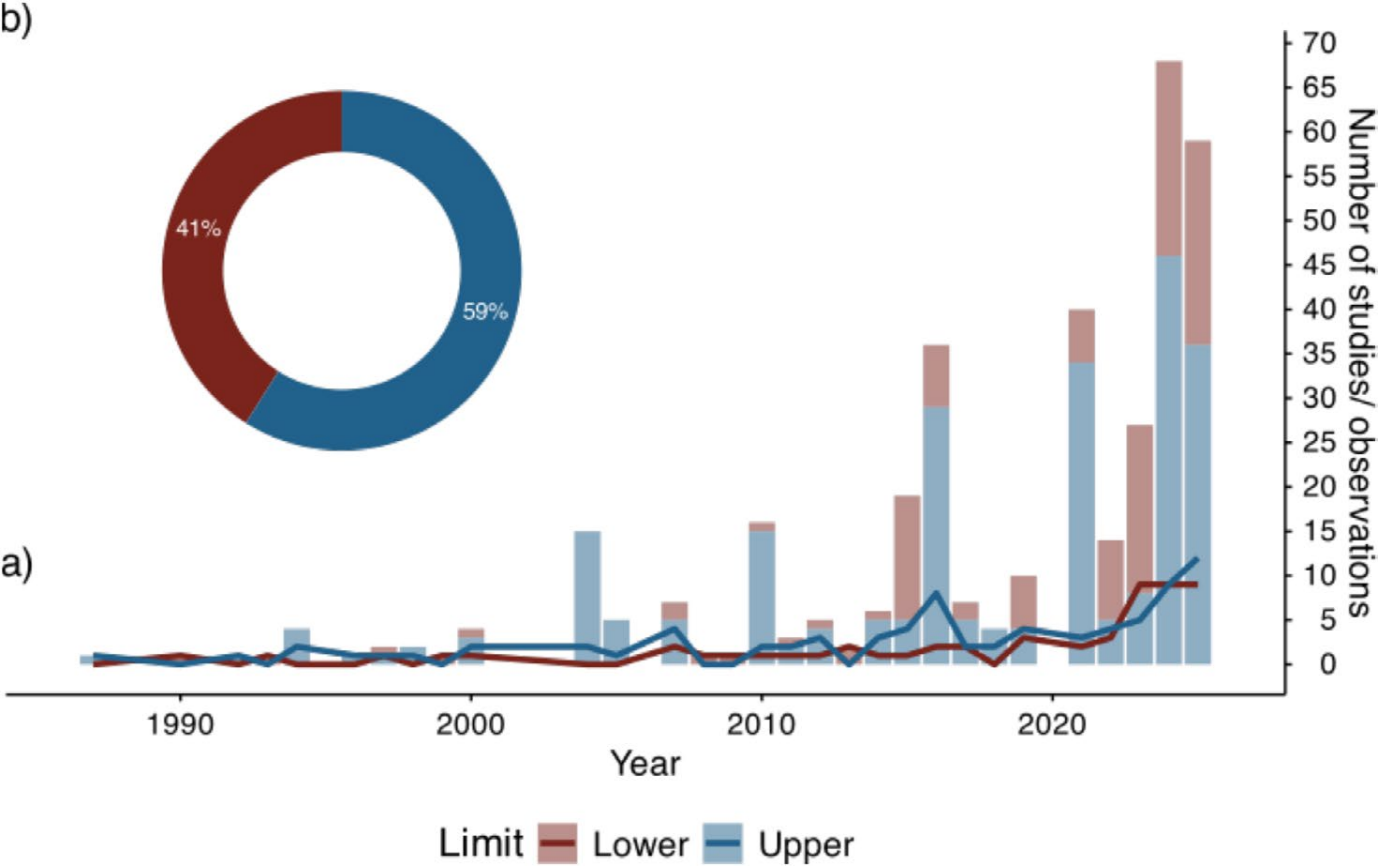
Research intensity of the upper and lower elevational range limits over time: (a) Number of studies (line graph) and observations (stacked bar plot) on the upper (blue) and lower (red) range limit of alpine plants over time. (b) Ratio of studies on the upper (blue) and lower (red) range limit per January 2026.

Most studies focused on the upper limits of alpine plant species: 79 studies (491 observations) addressed upper limits, compared to 55 studies (227 observations) focusing on lower limits. Of these, 27 studies (113 observations) actually focused on both limits. To improve clarity, observations from studies spanning both limits were included in both datasets. The skewed focus toward research on the upper range limit has been a general pattern over the last four decades, whereas this skew appears to be more pronounced when looking at the observations (Figure 2).

**FIGURE 3:**
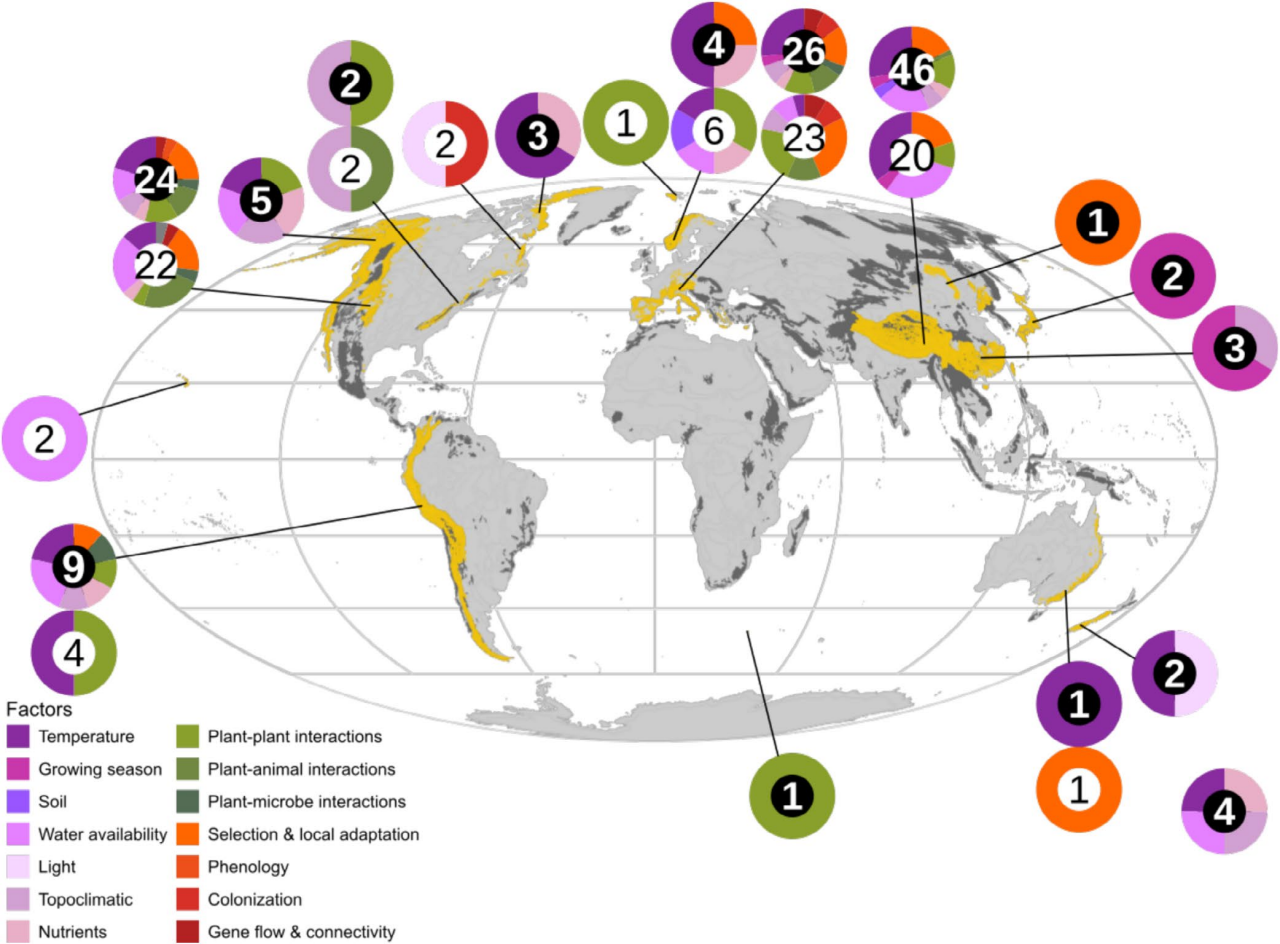
Global distribution of the 107 studies included in this review. Mountain ranges are depicted in dark gray and are highlighted in yellow if studies were located in them. The colored circles indicate which factors were investigated in each mountain range and the numbers in their centers the respective number of studies. Purple shades represent abiotic factors, green shades interspecific ones, orange shades represent intraspecific factors. White, bold numbers on black background refer to the upper range limit and black numbers on white background to the lower range limit. The figure positioned alone in the lower right corner represents worldwide studies.

**FIGURE A1:**
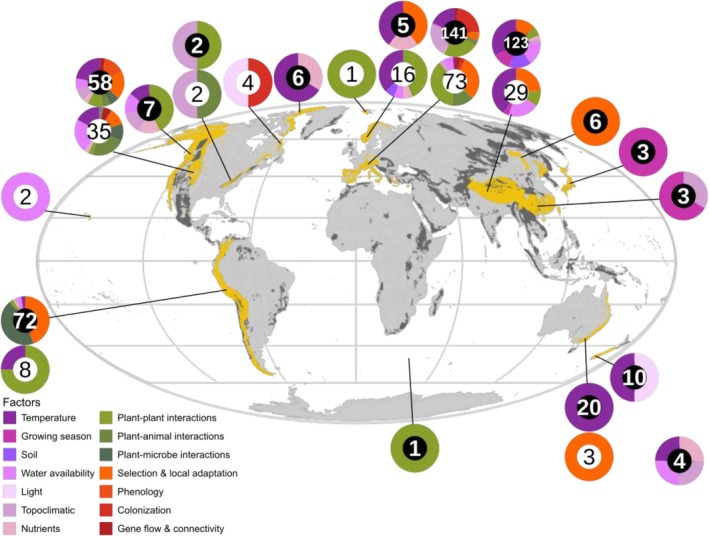
Global distribution of the observations included in this review. Mountain ranges are depicted in dark gray and are highlighted in yellow if studies were located in them. The colored circles indicate which factors were investigated in each mountain range and the numbers in their centers indicate the respective number of studies. Purple shades represent abiotic factors, green shades interspecific ones, orange shades represent intraspecific factors. White, bold numbers on black background refer to the upper range limit and black numbers on white background to the lower range limit. The figure positioned alone in the lower right corner represents worldwide studies.

**FIGURE A2:**
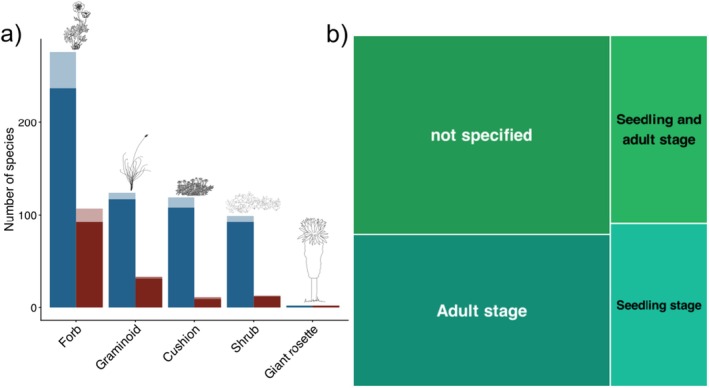
(a) Growth forms investigated for the upper (blue) and lower (red) elevational range limits and the number of species investigated for each growth form. Darker colors indicate species for which the original studies reported a statistically significant effect of the examined factor, while lighter colors represent cases where no statistically significant effect was reported. (b) Life stages of studied species for both range limits. The size of the boxes represents the number of species investigated for either adult stage, seedling stage, both life stages or if the study did not further specify the life stage.

In addition to the research bias toward the upper range limit, we detected a strong biogeographic bias in the scientific literature on elevational range limits. The large majority of studies (93%) were conducted in the Northern Hemisphere, dominated by studies in temperate climate regime (32% of all studies); only a few were in the Southern hemisphere (7%), and most of those were concentrated in a Mediterranean climate regime (3% of all studies). A very limited number of studies focused on the Tropics (4%), the Arctic (6%) and the boreal zone (6%). The vast majority of the studies was conducted in Central Europe, High Asia, and western North America (74%; Figure 3; for a breakdown of the same numbers focusing on observations, see Appendix C: Figure A1). Regarding life stages, a focus on adult individuals was evident (31% of the studies; 31% of observations), whereas the remainder (64% of studies; 69% of observations) either concentrated on seedlings (16%), on both seedlings and adult plants (21%), or did not specify the studied life stage (31%). In terms of growth forms, the currently available evidence is dominated by forbs (58%), which commonly represent the greatest fraction of alpine species (Körner 2021), followed by graminoids (26%), shrubs (22%), the special growth forms of cushion plants (18%) and giant rosettes (3%; see Appendix D: Figure A2).

### 3.1 Investigated Factors Behind Elevational Range Limits of Alpine Plant Species

**FIGURE A3:**
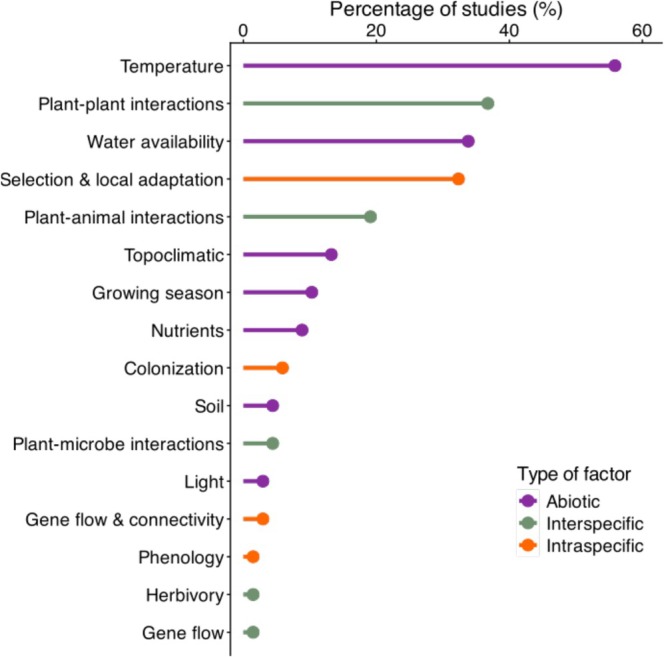
Relative frequency of studied elevational range limits. Percentage of the 107 studies in which the factors were assessed. Some studies focused on more than one factor and on both range limits, therefore having a higher impact in this figure.

Overall, abiotic factors were analyzed more frequently (54% of the studies) than interspecific factors (40%) or intraspecific factors (25%). Among the abiotic factors temperature alone was investigated in 36% of all studies (see Appendix E: Figure A3). Other abiotic factors included water availability (typically measured via precipitation or drought effects; 22%), topoclimatic factors (e.g., microhabitat variability, aspect or elevation; 8%), growing season length or onset (7%), nutrient availability (6%), soil characteristics (e.g., soil depth or development; 3%), and light (e.g., photoperiod or effects of shading; 2%). Among the interspecific factors, plant–plant interactions (e.g., facilitation, competition) were most commonly studied (23%), followed by plant–animal interactions (e.g., pollinator limitation, herbivory, nectar robbing; 12%) and plant–microbe interactions (e.g., plant–soil feedbacks; 3%). Intraspecific factors included selection and local adaptation (e.g., gene regulation and adaptations; 21%), colonization (i.e., dispersal and establishment; 4%) in the form of seed production, distribution and germination, as well as gene flow (e.g., as an assessment of heterosis; 3%) and phenology (e.g., timing of life‐history events such as flowering; 1%).

### 3.2 Investigated Factors at the Upper and Lower Elevational Range Limit

**FIGURE 4:**
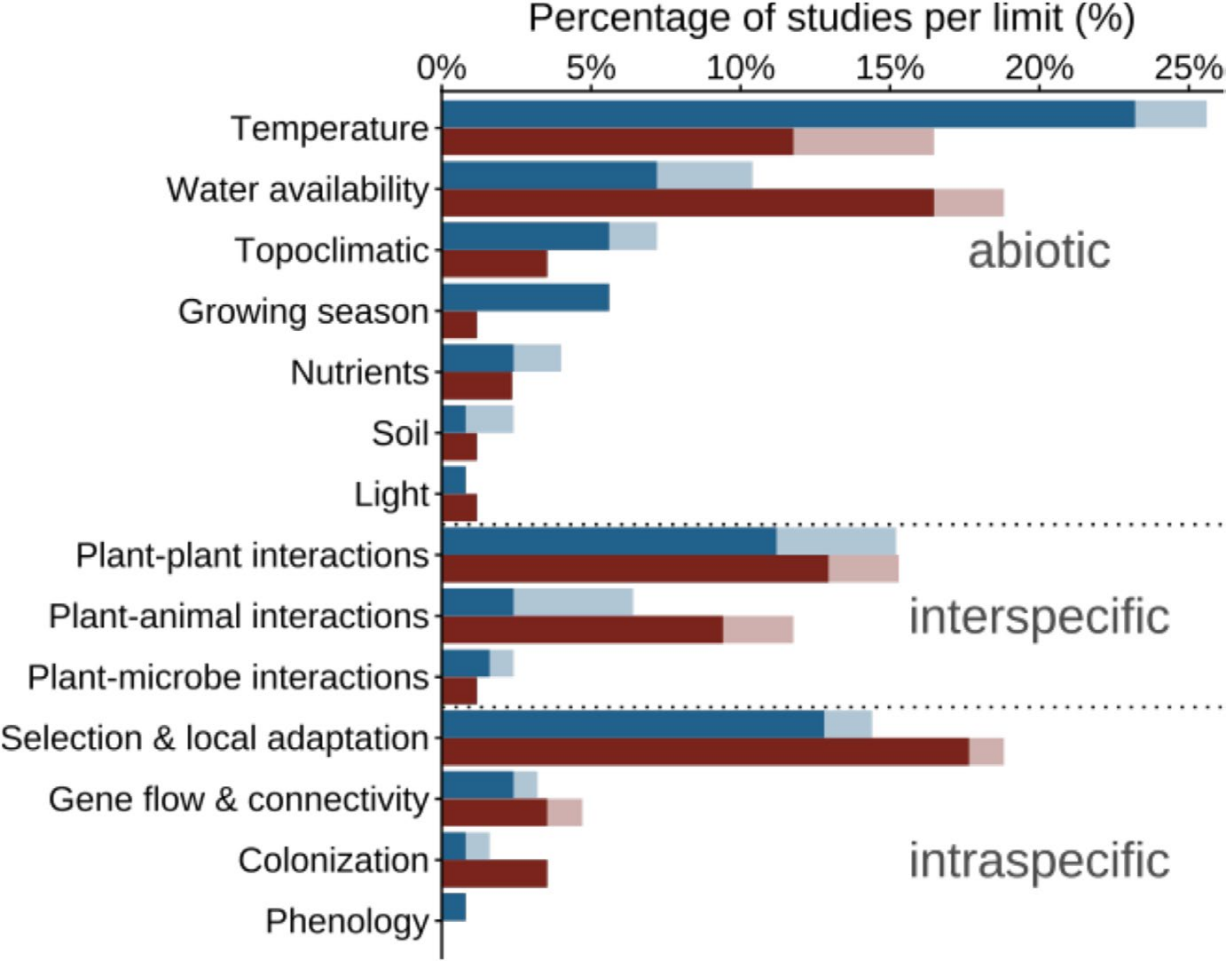
Factors of the upper (blue) and lower (red) range limit and percentage of studies per range limit. Dark colors within the bars represents the observations for which the factor was found to have an effect on range limits, light colors show where it was not. The dashed lines indicate the grouping of the factors in abiotic, interspecific and intraspecific factors.

**FIGURE A4:**
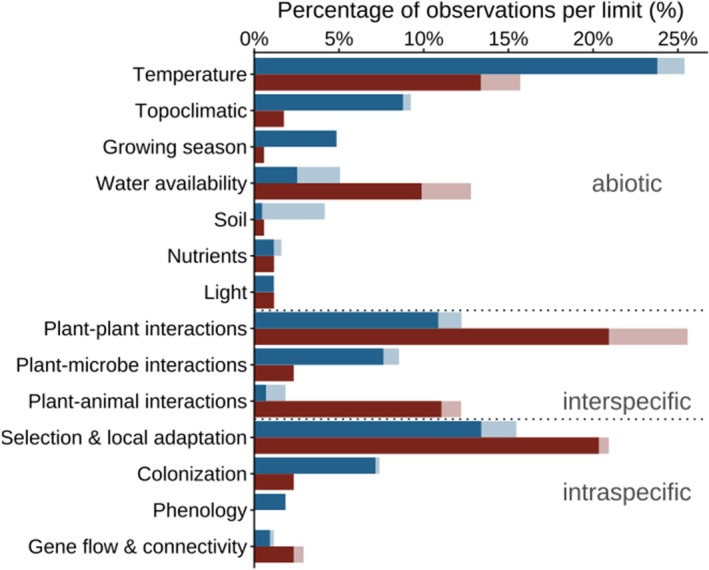
Factors examined at the upper (blue) and lower (red) elevational range limits, shown as the percentage of observations for each limit. Observations refer to unique species–factor combinations within studies. Dashed lines indicate the grouping of factors into abiotic, interspecific, and intraspecific categories.

The factors which were most commonly studied at the upper and lower elevational range limits differed. At the upper limit, temperature was the most studied factor (29% of the studies, 18% of observations), whereas most studies at the lower limit investigated water availability (15% of the studies; Figure 4) and most observations were interpreted in terms of selection & local adaptation (12% of observations; see Appendix F: Figure A4). In general, abiotic factors have been studied more on the upper limit and inter‐ and intraspecific ones at the lower limit (Table 1, Figure 4).

**TABLE 1:** Factors influencing the elevational range limits of alpine plant species.

	Expected factor	Upper limit	Lower limit
Abiotic	Growing season	*Snowmelt date* (Kawai and Kudo 2018; Zong et al. 2022; Li et al. 2025), *snow cover* (Carrer et al. 2019), *growing season start and length* (Maruta 1994; Klimeš and Doležal 2010; Chen et al. 2024), *extreme events in the growing season* (Chen et al. 2024)	G*rowing season length and extreme events in the growing season* (Chen et al. 2024)
	Topo‐climatic	**Microsite availability** (García‐Camacho et al. 2010), **special microhabitats within suitable habitats** (Aikens and Roach 2014), **rugged topography (temperature modulation, retention of soil organic matter)** (Spasojevic et al. 2014), *mountain height, wind speed* (Zou et al. 2023), habitat suitability (based on elevation, substrate characteristics, slope, solar radiation, aspect, exposure, terrain curvature, terrain ruggedness and topographic wetness; Helm et al. 2024), elevation (Büntgen et al. 2025; Yang et al. 2025)	Microhabitat/‐site variability (García‐Camacho and Escudero 2009) and availability (García‐Camacho et al. 2010), special microhabitats within suitable habitats (Aikens and Roach 2014)
	Light	Relation of daylength with frost resistance (Bannister et al. 2005)	*Shading* (Haynes et al. 2021)
	Nutrients	*Nutrient uptake limitation* (*by poorly developed soil, scarce water, low soil temperature*; Volder et al. 2000; Macek et al. 2012), *lack of nutrients* (Diemer 1992; Theodose and Bowman 1997; Zou et al. 2023; Mucha et al. 2025)	*Nutrients* (Theodose and Bowman 1997), *lack of nutrients* (Mucha et al. 2025)
	Soil	*Substrate availability, feedback or composition* (Klimeš and Doležal 2010; White et al. 2024)	*Low soil carbon* (Mucha et al. 2025)
	Temperature	*Freezing* (Lambrecht et al. 2007; Yi et al. 2011; Wang et al. 2021), *low temperature* (Perez 1987; Atkin and Cummins 1994; Pittermann and Sage 2000; Ruotsalainen and Kytöviita 2004; Bannister et al. 2005; Klimeš and Doležal 2010; Boucher et al. 2012; Dvorský et al. 2016; Meng et al. 2016; Nagelmüller et al. 2016, 2017), *soil minimum temperature in winter* (von Büren and Hiltbrunner 2022), *temperature variation according to slope orientation and aspect* (Diemer 1992; Cuena‐Lombraña et al. 2016), *low air temperature in the growing season* (Li et al. 2016), *temperature thresholds, frost sensitivity* (CaraDonna and Bain 2016) *and resistance* (Taschler and Neuner 2004), *early season temperature and snow cover* (Carscadden et al. 2023), *mean temperature in winter* (Zou et al. 2023), *frost events in winter* (Dolezal et al. 2016; White et al. 2024), *low threshold in soil temperature* (Hirst et al. 2025), *diurnal temperature fluctuations in summer* (Dolezal et al. 2016)	*Temperature thresholds* (Meng et al. 2016), *temperature fluctuations, freezing* (Lambrecht et al. 2007), *low temperatures* (Han et al. 2022), *warm temperatures* (Cavieres and Arroyo 2000; Cui et al. 2017; Oldfather and Ackerly 2019), *high early‐season temperatures* (Carscadden et al. 2023), *heatwaves* (Bonanomi et al. 2023), warming (Cavieres et al. 2024; Thakur et al. 2025)
	Water availability	*Low (soil) moisture* (Perez 1987; Theodose and Bowman 1997; Lambrecht et al. 2007; Han et al. 2022), *drought stress* (Dvorský et al. 2016; Wang et al. 2021), *climatic water deficit, evapotranspiration* (Zou et al. 2023), *summer floods* (Dolezal et al. 2016), *drought resulting in reduced snow cover* (White et al. 2024)	*Drought stress* (Giménez‐Benavides et al. 2008; Krushelnycky et al. 2013; Han et al. 2022), *low soil moisture* (Theodose and Bowman 1997; Lambrecht et al. 2007; Yi et al. 2011; Cui et al. 2017), *insufficient precipitation in the dry season* (Berio Fortini et al. 2022), *trade‐off between drought tolerance and defense against herbivores* (Alsdurf et al. 2013), **sufficient water‐availability** (Oldfather and Ackerly 2019), *trade‐off between warming and competition* (Nomoto et al. 2024)
Interspecific	Gene flow	**Heterosis (hybrid expands beyond range limits of parents**; Boller et al. 2023; Carscadden et al. 2023)	**Heterosis (hybrid expands beyond range limits of parents**; Boller et al. 2023; Carscadden et al. 2023)
	Plant–animal interactions	*Nectar robbing and flower predation* (Kohl and Steffan‐Dewenter 2022), *herbivory* (Diemer 1996), *pollinator decline* (Hargreaves et al. 2015), *pollinator availability* (Theobald et al. 2016; Kohl and Steffan‐Dewenter 2022; Villa et al. 2025), *reduced pollinator service* (Moeller et al. 2012), *grazing and disturbance* (Yang et al. 2025)	*Herbivory* (Galen 1990; Giménez‐Benavides et al. 2008; Lynn et al. 2021; Bovay et al. 2025), *flower predation* (Giménez‐Benavides et al. 2008), *trade‐off between herbivory defense and drought tolerance* (Alsdurf et al. 2013), *specialized pollinators* (Garcia et al. 2023), *pollinator decline* (Theobald et al. 2016), *reduced pollinator service* (Moeller et al. 2012), *attractiveness to pollinators* (Garcia et al. 2023), **pollinator availability** (Villa et al. 2025)
	Plant–microbe interactions	**Positive plant–soil feedback** (Sedlacek et al. 2014), **recruitment of beneficial microbes** (Eshel et al. 2021), *negative plant–soil feedback* (Luo et al. 2025)	*Negative plant–soil feedback* (Luo et al. 2025)
	Plant–plant interactions	**Facilitation by neighboring plants (e.g., cushion plants and willows) which buffer temperature extremes, lower the macroclimatic minimum growing season temperature, reduce herbivory, reduce fungal infections, provide higher soil organic content, enhance soil moisture and lower evaporation rates, provide shade, wind blockage and snow retention, nurse‐plant effect** (Wied and Galen 1998; Cavieres et al. 2007; Dona and Galen 2007; de Bello et al. 2011; Spasojevic et al. 2014; Pugnaire et al. 2015; Wheeler et al. 2015; Raath‐Krüger et al. 2019; Ale et al. 2023; Wang et al. 2024; Yang et al. 2025), *competition with other plants* (e.g., *dwarf shrubs, bigger plants*; Theodose and Bowman 1997; Angers‐Blondin et al. 2018; Crepaz et al. 2021; Chen et al. 2024; Helm et al. 2024)	*Competition*, e.g., *for light, nutrients, water* (Havström et al. 1993; Theodose and Bowman 1997; Flegrová and Krahulec 1999; Lyu and Alexander 2022, 2023; Schuchardt et al. 2023; Chen et al. 2024), *trade‐off between competition and drought adaptation* (Nomoto et al. 2024), **facilitation by neighboring plants which enhance moisture and reduce radiation** (Ale et al. 2023; Cáceres‐Mago et al. 2023; Cavieres et al. 2024), **competitive advantage** (Dahle et al. 2024)
Intraspecific	Colonization	*Dispersal ability* (Helm et al. 2024), *seed availability* (García‐Camacho et al. 2010)	*Low seed production in pre‐dispersal stage* (Giménez‐Benavides et al. 2008), *seed availability* (García‐Camacho et al. 2010), *niche limits in seed emergence* (Haynes et al. 2021)
	Gene flow and connectivity	**Gene flow between populations maintains genetic exchange** (Felkel et al. 2023)	**Gene flow between populations maintains genetic exchange** (Felkel et al. 2023)
	Phenology	**Seasonal frost resistance, temperature sensitivity of phenophases** (CaraDonna and Bain 2016)	—
	Selection and local adaptation	**Local adaptations, e.g., via high/low plasticity** (Giménez‐Benavides et al. 2007; Halbritter et al. 2015; McIntyre and Strauss 2017; Hargreaves and Eckert 2019; Eshel et al. 2021; Felkel et al. 2023; Daco et al. 2024; Doležal et al. 2024; Du et al. 2024; Luo et al. 2024; Chen, Jia, et al. 2025; Kumari et al. 2025; Narasimhan and Willi 2025), **upregulation/expression of genes in range edge populations** (Gurung et al. 2019; Eshel et al. 2021; Mangral et al. 2025; Tisinai et al. 2025), **frost sensitivity as a result of evolutionary history** (CaraDonna and Bain 2016)	**(Local) adaptations** (Giménez‐Benavides et al. 2007; McIntyre and Strauss 2017; Hargreaves and Eckert 2019; Daco et al. 2024; Du et al. 2024; Felkel et al. 2023; Chen, Jia, et al. 2025; Narasimhan and Willi 2025; Vázquez‐Ramírez et al. 2025), **upregulation/expression of genes in range edge populations** (Gurung et al. 2019; Tisinai et al. 2025), *adaptation to upper range limit results in maladaptation at the lower limit* (Gugger et al. 2015; Walter et al. 2024), *lack of adaptive response* (Schuchardt et al. 2023)

*Note:* Factors are grouped by their type (abiotic, interspecific, intraspecific), the range limit (upper and lower) and the description of the factor as reported in the studies included in this review: Bold text denotes stabilizing effects of the factor at the range limit, defined as factors that facilitate persistence at the limit or whose absence would lead to range contraction, while italic text denotes a restrictive effect, defined as factors imposing a constraint on persistence at the range limit, and empty fields indicate that no studies were found within the scope of this review. The color of the fields marks the type of factor: Purple for abiotic factors, green for interspecific factors and orange for intraspecific factors.

Of the 14 factors considered in this review, only growing season length (7 studies, 22 observations), phenology (1 study, 8 observations) and light (e.g., photoperiod or effect of shading; 2 studies, 7 observations) were reported to affect elevational range limits in every study and species in which they were examined, although this pattern is based on a very limited number of studies for phenology and light. In contrast, soil characteristics were identified as the determining factor in less than 50% of the observations (16%; Figure 4; for information on observations see Appendix F: Figure A4). Together, this indicates that, all factors identified in this review were relevant under at least some conditions, highlighting strong context dependency of factors determining elevational range limits.

Within studies, the factor in focus was often examined in multiple contexts (e.g., pollinator limitation and nectar robbing, both summarized as plant–animal interactions), yet only 36% of the 107 studies investigated more than one distinct factor simultaneously, revealing a strong bias toward single‐factor approaches. Although all factors might shape elevational range limits, some are constraining whereas others are stabilizing. For example, cold temperatures typically constrain upper range limits, while plant–plant interactions can either stabilize (e.g., facilitation) or constrain the limits (e.g., competition). Local adaptation may increase plant tolerance to abiotic stress but might entail trade‐offs that limit performance in another way. Table 1 summarizes the reported constraining or stabilizing effects of each factor in the reviewed studies.

### 3.3 Specifications of Temperature‐Related Variables

**FIGURE 5:**
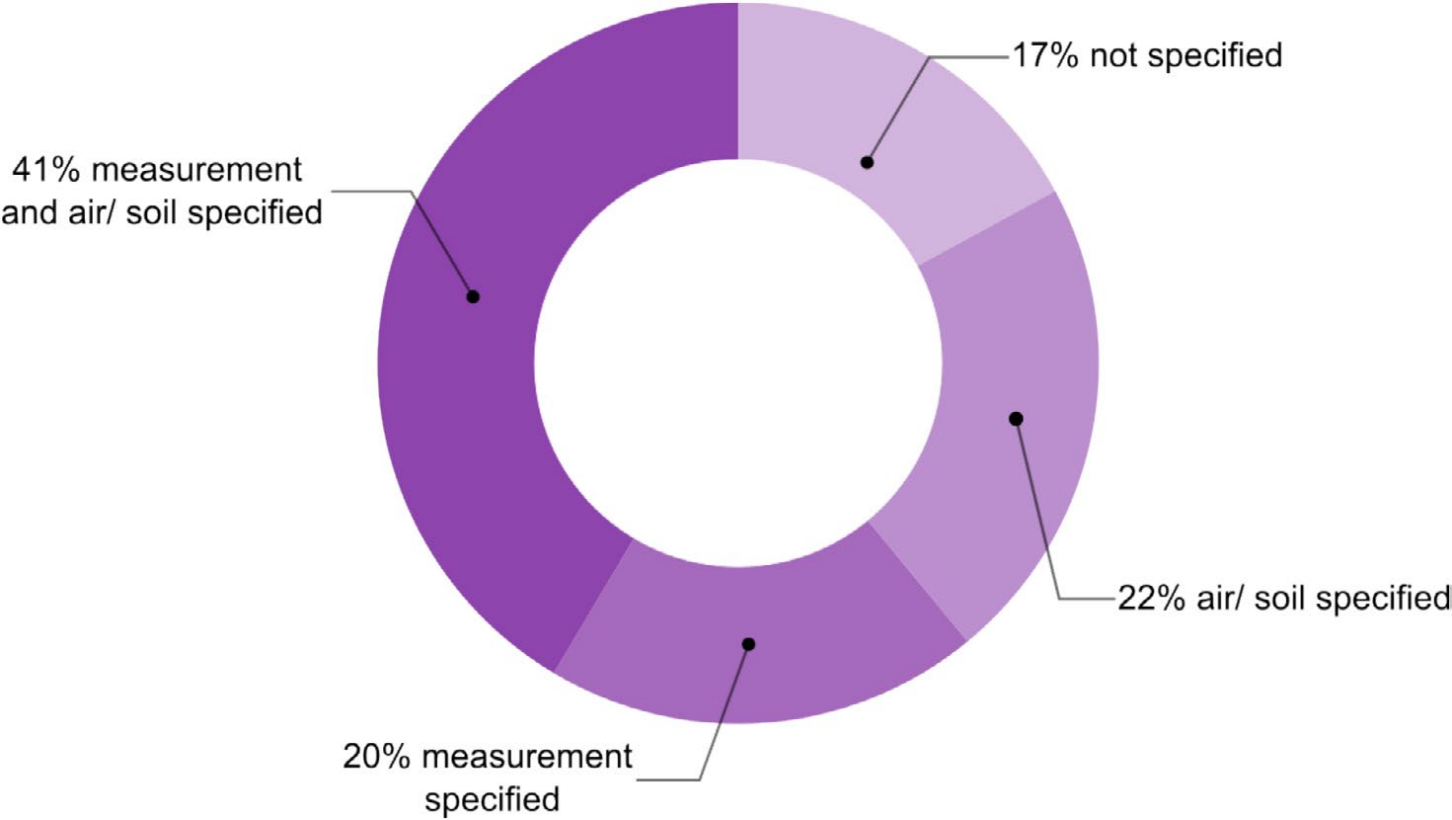
Details on temperature measurements reported by 41 studies. The category “not specified” included seven studies that did not specify whether air or soil temperature was measured, nor when and how measurements were taken (e.g., “temperature by slope and aspect”). In the category “air/soil specified,” nine studies reported whether air or soil temperature was measured but did not specify the temperature metric used (e.g., “cold air temperature”). The category “measurement specified” comprises eight studies that reported only the temperature metric used but did not indicate whether measurements referred to air or soil temperature (e.g., “minimum temperature in winter”). Only seventeen studies fell into the category “measurement and air/soil specified”, reporting both whether soil or air temperature was measured and which temperature measure was used (e.g., “minimum soil temperature in winter”).

Temperature was the most frequently studied factor hypothesized to influence the range limits of alpine plants. Accordingly, the question which aspect of temperature is relevant for the distribution of alpine plant species has received increasing attention in recent years (Körner and Hiltbrunner 2018; Lembrechts et al. 2019). While temperature was clearly deemed to be important at the upper range limit given the number of studies (Figure 4), studies varied widely in how minimum temperature was defined. Reported metrics included mean or minimum soil or air temperature during the growing season (Li et al. 2016; Nagelmüller et al. 2017; Han et al. 2022), absolute minimum temperature (von Büren and Hiltbrunner 2022), and freezing events during the growing season (Lambrecht et al. 2007). However, only 22% of the studies specified whether air or soil temperature was used and not which metric was assessed, and 20% of the studies did not specify whether the measurements they reported referred to soil or air temperature (Figure 5).

## 4 Discussion

By synthesizing 107 studies published over the past 39 years, this review suggests that elevational range limits emerge from the interaction of multiple processes, including abiotic constraints, biotic filtering, dispersal, demographic dynamics, and evolutionary responses—and that this complex causation likely differs between upper and lower range limits. Nevertheless, the research effort so far has not been evenly distributed across factors and range limits, but directed largely toward upper range limits—with roughly one‐and‐a‐half times as many studies as for lower limits—and toward abiotic factors, particularly temperature.

### 4.1 Missing and Underrepresented Factors

Research on the range limits of alpine plants has so far strongly focused on low temperatures, short growing seasons together with topo‐climatic proxies that are commonly used to represent temperature and growing season length at upper limits. This emphasis likely reflects early hypotheses posting cold temperature to be the major limiting factor of the upper range limit (Darwin 1859; MacArthur 1972) and, more recently, the practical availability of macroclimatic datasets that facilitate broad‐scale analyses (e.g., Karger et al. 2017). By comparison, studying inter‐ and intraspecific factors often requires more resource‐intensive, longer‐term, and individual‐based approaches (Körner 2021), and some factors could not be studied at all until recently (e.g., metagenomic studies). As a result, emerging areas such as plant–microbiome interactions and the impact of pathogens, theoretically predicted to be influential (Paquette and Hargreaves 2021), are almost entirely absent from the empirical literature. Beyond these limitations, three factors are particularly underrepresented. First, dispersal limitation: many alpine plants have short dispersal distances (Morgan and Venn 2017; Tovar et al. 2020), transient seed banks (Jaganathan et al. 2015), and few dispersal vectors (Tovar et al. 2020), constraining range limits even where conditions seem otherwise suitable (Helm et al. 2024). Second, life‐stage bottlenecks and population dynamics: germination, establishment, and juvenile survival may be more restrictive at the range limit than adult survival (Maruta 1994; Tudela‐Isanta et al. 2018; Lin et al. 2023) and are also often the limiting factor in the responses of alpine species to climate warming (e.g., shrub recruitment fails under climate warming; Büntgen et al. 2015; Lu et al. 2022). Yet, most studies (39%) have focused on adult individuals or did not distinguish between life stages. Third, disturbance regimes such as extreme frost events and trampling (Chardon et al. 2019), grazing (Lin et al. 2023) as well as other human disturbances such as species removal (Yang et al. 2025) shape microsite availability in some regions and mediate both abiotic stress and species interactions, but these are rarely quantified.

### 4.2 Consequences of Missing Factors for Our Understanding of Elevational Range Limits

Macro‐temperature is readily available (e.g., Karger et al. 2017) and a key driver of range shifts, yet it leaves much variation of range limits (Andrello et al. 2020) and range shifts (Lawlor et al. 2024) unexplained. Several of the studies in this review show that range limits seldom depend on a single factor. Abiotic stress often intensifies interspecific interactions (especially facilitation at the upper and competition at the lower range limit; Liancourt et al. 2017), local adaptations in range limit populations enable coping with stressors but entail trade‐offs [e.g., between drought tolerance and herbivory resistance (Alsdurf et al. 2013) or between responses to warming and competition (Nomoto et al. 2024)], and facilitation can buffer multiple stressors simultaneously (Wheeler et al. 2015). Further, the importance of factors might change under climate warming, as other factors, such as water limitation, become limiting (Dolezal et al. 2020). As emphasized below, future research should pay greater attention to assessing both abiotic and inter‐ and intraspecific biotic factors as well as their interactions in modulating elevational range limits.

### 4.3 Limitations and Caveats, Measurements and Reporting

Factors affecting the lower range limit of plants have been underexplored, both regarding the position of range limits and range shifts (Lenoir and Svenning 2015). This (apparent) paucity of studies is likely partly a result of how often the lower range limit lies below the treeline. Although we included cases where an alpine species' lower limit was below treeline, studies conducted entirely below treeline that did not flag alpine taxa in the title or abstract may have been missed in this review. Consequently, lower range limits may involve factors typical of subalpine or montane systems (e.g., competition with woody plants, land use changes) rather than uniquely alpine factors. Geographic biases could further impair the generalizability of the here detected factors. For example, water availability emerges as a limiting factor, but primarily in already water‐limited systems such as the Mediterranean (Giménez‐Benavides et al. 2008).

We are gradually gaining an overview of the key factors that shape elevational range limits. The next step should be to gain a more mechanistic understanding of how these factors exert their effects, and which specific aspects of these factors matter. For instance, although temperature consistently emerges as an important factor, it remains uncertain which aspects of temperature, such as the medium (e.g., air or soil temperature), timing (e.g., temperature during growing season or winter), measurement method (e.g., weather‐station data, in situ loggers), or metric (e.g., mean, minimum, or maximum temperature) are most relevant (see the review by Körner and Hiltbrunner 2018). To address this limitation, future studies should report in detail, where, when, and for how long temperature as well as any other factor was measured, because these aspects likely capture distinct ecological processes influencing range limits. Furthermore, improved specification of the factors would improve the predictive performance of species distribution models and thereby enhance our ability to predict the impacts of climate change. Equally important is the consistent reporting of effect sizes and uncertainty which would also bolster comparability and allow for quantitative syntheses across studies (Gerstner et al. 2017). An open database compiling information on species ranges and the factors stabilizing and constraining them would provide a valuable resource for the planning of future research, including studies on the potential and already apparent effects of climate change. Finally, positive publication bias, together with financial and logistical constraints, entail methodological biases toward short‐term studies of adult individuals in well‐studied mountain systems, further limiting generalizations across life stages, timescales, and regions. These limitations pose particular risks when such evidence is used to inform projections and management decisions under climate change. In particular, conclusions derived from a narrow subset of species, life stages, or abiotic factors may result in misleading recommendations, for example, when used as the basis for the designation of protected areas (Velazco et al. 2020).

### 4.4 Future Research Directions

Under climate change, cold temperature constraints at upper range limits might diminish, thereby making research on other factors even more important. Although temperature is a fundamental factor, it is unlikely to fully explain elevational range limits on its own. Understanding the processes governing the range limits of alpine plants requires moving beyond temperature to capture the multi‐factor processes that jointly set range limits. We propose three emerging priorities. (i) Integrative experiments and models: future studies should combine abiotic with inter‐ and intraspecific interactions, such as water availability with plant–soil feedbacks or local adaptations. Multifactor experiments and mechanistic distribution models (process‐based models that predict species distributions from physiological and demographic constraints, not correlations) are essential to explain range limits. (ii) Demography and evolutionary capacity: key life‐history transitions such as germination, establishment, and seedling survival remain underexplored, despite their potential to define range limits more strongly than adult survival. Linking these stages with local adaptation and genetic drift would also clarify the capacity of alpine plants to cope with changing climates. (iii) Removing geographic biases by expanding the focus toward the Tropics, the Arctic, and the Southern hemisphere. Because most studies originate from temperate Northern Hemisphere mountains, the factors highlighted in this review may disproportionately reflect processes characteristic of these regions rather than universal drivers of alpine range limits. Expanding research across other latitudes and climatic zones will be important for assessing the generality of these patterns.

## Author Contributions

**Sophie E. Weides:** conceptualization (lead), data curation (lead), formal analysis (lead), investigation (lead), methodology (lead), visualization (lead), writing – original draft (lead), writing – review and editing (lead). **John‐Arvid Grytnes:** conceptualization (supporting), supervision (supporting), visualization (supporting), writing – review and editing (supporting). **Stefan Dullinger:** conceptualization (supporting), writing – review and editing (supporting). **Jonathan Lenoir:** conceptualization (supporting), writing – review and editing (supporting). **L. Camila Pacheco‐Riaño:** conceptualization (supporting), visualization (supporting), writing – review and editing (supporting). **John R. Pannell:** conceptualization (supporting), writing – review and editing (supporting). **Sonja Wipf:** conceptualization (supporting), writing – review and editing (supporting). **Sabine B. Rumpf:** conceptualization (equal), formal analysis (supporting), funding acquisition (lead), methodology (equal), supervision (lead), visualization (supporting), writing – review and editing (supporting).

## Conflicts of Interest

The authors declare no conflicts of interest.

## Supporting information


**Data S1:** ece373183‐sup‐0001‐supinfo.R.

**Table S1:** Studies included in the literature review. This table provides the full list of the 107 studies included in this review, including basic study information such as authors, publication year, study location and biome, number of species studied and their names, life stages and growth forms, research approach, the elevational range limit examined, the factors investigated, and the reported effects of these factors on the range limits.

## Data Availability

All data used are available in the Supporting Information.

## Appendix A

### Boolean Strings for the Literature Search

The following Boolean strings were used for Web of Science and for Scopus. Both have the exact same criteria, but differ slightly in syntax.

1. Boolean string for Web of Science

((TI=((elevat* OR altitud* OR alpin* OR montan* OR nival* OR mountain*) AND (distribut* OR range* OR niche* OR gradient* OR shift* OR occurrence* OR abundance* OR location* OR adaptation* OR expan* OR retract* OR grow* OR surv* OR reproducti* OR germinati* OR speciati* OR evoluti*) AND (limit* OR edge* OR margin* OR boundar* OR border* OR constraint* OR restrain* OR restrict* OR confin* OR extrem* OR verge* OR brink* OR threshold*) AND (plant* OR vegetat* OR shrub* OR grass* OR sedge* OR herb* OR forb* OR graminoid* OR cushion*))) OR AB=((elevat* OR altitud* OR alpin* OR montan* OR nival* OR mountain*) AND (distribut* OR range* OR niche* OR gradient* OR shift* OR occurrence* OR abundance* OR location* OR adaptation* OR expan* OR retract* OR grow* OR surv* OR reproducti* OR germinati* OR speciati* OR evoluti*) AND (limit* OR edge* OR margin* OR boundar* OR border* OR constraint* OR restrain* OR restrict* OR confin* OR extrem* OR verge* OR brink* OR threshold*) AND (plant* OR vegetat* OR shrub* OR grass* OR sedge* OR herb* OR forb* OR graminoid* OR cushion*))) OR AK=((elevat* OR altitud* OR alpin* OR montan* OR nival* OR mountain*) AND (distribut* OR range* OR niche* OR gradient* OR shift* OR occurrence* OR abundance* OR location* OR adaptation* OR expan* OR retract* OR grow* OR surv* OR reproducti* OR germinati* OR speciati* OR evoluti*) AND (limit* OR edge* OR margin* OR boundar* OR border* OR constraint* OR restrain* OR restrict* OR confin* OR extrem* OR verge* OR brink* OR threshold*) AND (plant* OR vegetat* OR shrub* OR grass* OR sedge* OR herb* OR forb* OR graminoid* OR cushion*))

2 Boolean string for Scopus

TITLE‐ABS‐KEY = ((elevat* OR altitud* OR alpin* OR montan* OR nival* OR mountain*) AND (distribut* OR range* OR niche* OR gradient* OR shift* OR occurrence* OR abundance* OR location* OR adaptation* OR expan* OR retract* OR grow* OR surv* OR reproducti* OR germinati* OR speciati* OR evoluti*) AND (limit* OR edge* OR margin* OR boundar* OR border* OR constraint* OR restrain* OR restrict* OR confin* OR extrem* OR verge* OR brink* OR threshold*) AND (plant* OR vegetat* OR shrub* OR grass* OR sedge* OR herb* OR forb* OR graminoid* OR cushion*))

### Specification of Inclusion and Exclusion Processes of Studies

In total, we conducted two literature searches. The first one on December 1, 2022 and the second one on January 15, 2026. In both cases, the same Boolean string was applied (Appendix A.1). The first search yielded 30,696 studies, while the second yielded 5591 studies.

The two searches only differed in the screening procedure applied during the initial automated screening step. For the first search, a less elaborate automated screening approach was used. By the time of the second search, increased familiarity with the types of records retrieved allowed us to refine and expand the automated screening criteria, thereby improving the efficiency of record exclusion.

In the first search (until 2022) any record was excluded whose title contained case‐insensitive matches to an exclusion string comprising terms related to (i) crops/agronomy and managed production systems (crop, wheat, cereal, maize, rice, cotton, tomato, soy, potato, coffee, rapeseed, cauliflower, *Brassica napus* , chickpea, lettuce, lentil, pumpkin, agroforest, farming, livestock, agricultur), (ii) aquatic, marine, or saline contexts (ocean, lake, sea, marin, salin, saltmarsh, aquatic, salt, wetland, freshwater, reef, seagrass, coral), and (iii) other non‐target topics (paleo, archeology, parasit, host, carnivor, sahara, urban, mercury, electricity). This excluded 4156 studies from the review.

In the second search, papers whose titles contained case‐insensitive matches to terms referring to specific genera, species, or forest types (Nothofagus, *Betula pendula* , *Betula pubescens* , Fagus, European beech, *Fagus sylvatica* , *Alnus glutinosa* , beechwood, beechwoods), geographic regions (Negev), paleontological or proxy‐based studies (fossil, fossils, tree rings), non‐target organism groups (baboons, Culicoides, bryophyte, bryophytes), domesticated or cultivated plants (crop, crops, domesticated, fig, figs, avocado, avocados), and aquatic plant systems (aquatic vascular plant, aquatic vascular plants) were excluded. We further removed dataset‐type records using a dataset‐indicator pattern (“data for/from…,” “data sheet,” “dataset”) and excluded records whose source field corresponded to major data repositories (Zenodo, Dryad, Figshare). We then filtered out remaining non‐target studies by excluding any record whose title or abstract contained crop‐related terms (wheat, rice, maize, corn, soybean, soya, barley, oat, rye, sorghum, millet, potato, sweet potato, cassava, yam, taro, tomato, cucumber, pepper, capsicum, eggplant, aubergine, onion, garlic, leek, lettuce, cabbage, broccoli, cauliflower, spinach, kale, carrot, beet, radish, pea, beans?, chickpea, lentil, apple, grape, citrus, orange, lemon, lime, banana, mango, strawberry, blueberry, coffee, cocoa, cacao, tea, olive, canola, rapeseed, sunflower, peanut, groundnut, cotton, sugarcane, sugar beet, oil palm) or marine/coastal and saltwater terms (marine, ocean, sea, saltwater, saline, brackish, coastal, estuar(y|ine), lagoon, tidal, intertidal, salt marsh, marsh, mangrove, seagrass, kelp, seaweed, macroalga(e), coral, reef, fish, fishes, shellfish, crab, shrimp, prawn, oyster, mussel, clam, aquaculture, fisher(y|ies|ing)). Finally, because our target system was non‐forest terrestrial ecology, we excluded papers with forest in the title unless they explicitly referred to boundary contexts (e.g., ecotone(s), treeline/tree line, timberline, forest–grassland/forest grassland), and we removed records mentioning treeline‐associated conifer/gymnosperm taxa in the title or abstract (pinus/pine(s), picea/spruce(s), abies/fir(s), larix/larch(es), juniper(us), cedrus/cedar(s), pseudotsuga, douglas fir/douglas‐fir, cupressus/cypress(es), taxus/yew(s), conifer(s), gymnosperm(s)). This excluded 833 studies from the review.

All subsequent steps and inclusion/exclusion criteria were applied consistently across both searches.

In regard to quality assessment, we rely on peer review in respected journals. Therefore, we excluded journals classified as predatory by the webpage https://www.predatoryjournals.org, which is curated by an anonymous organization of volunteer researchers.

**TABLE A1:** Classification of extracted information from the 107 studies included in the literature review. Category, exact information extracted, and explanation of extracted information for the 100 studies included in this review. See Table S1 for the categorization of each study.

Level of information	Category	Explanation
Study‐specific	Author	Author(s) of the study
	Year	Year the study was published in
	Biome	Biome of the alpine zone the study was conducted in (after Olson et al. 2001): Arctic and boreal/temperate/winter‐cold dryland/Mediterranean/subtropics and tropics/worldwide
	Location of study	Mountain range the study was conducted in, as indicated in the publication
	Number of species	Number of plant species investigated/assemblage/family/genus The term assemblage is used for a group of species that is not further specified.
	Research approach	Model: modeling approaches (e.g., species distribution modeling). Observation: observations in the field Experiments: lab or field experiments
	Limit	Upper range limit, lower range limit, or both limits
Factor‐specific	Factor	An overview of the factors is given in Section 4
	Multiple Factors	One or more factors investigated: Yes/No
	Importance	If multiple factors were investigated, their individual importances were classified as Equal: investigated factors were equally important in determining the range limit Higher: one factor was more important than the other factor(s) Lower: one factor was less important than the other factor(s)
	Effect of factor	Yes: A factor was identified as determining for the range limit No: A factor was either found to have no effect or to only have an effect if the main factor was not limiting (e.g., temperature is only limiting when soil moisture is sufficient)
Species‐specific	Species	Scientific name of the focal species
	Growth form	Forb/graminoid/cushion/rosette/shrub/not specified. If plant assemblages, genera or families were studied, growth form was not specified
	Life stage	Seedling/adult/seedling and adult/not specified

## Appendix B

### Species Occurrences in Studies

**TABLE A2:** Number of studies each species occurs in.

Number of different studies the species occurred in	Number of species	Species name(s)
6	2	*Poa alpina, Ranunculus glacialis*
5	1	*Plantago alpina*
4	1	*Anthyllis vulneraria* ssp. *alpestris*
3	5	*Boechera stricta, Poa attenuata, Sesleria caerulea, Silene acaulis, Trifolium badium*
2	23	*Androsace alpina, Anthoxanthum alpinum, Argyroxyphium sandwicense* subsp. *macro‐cephalum, Armeria caespitosa, Arnica montana, Aster alpinus, Campanula scheuchzeri, Cardamine resedifolia, Carex curvula, Cerastium uniflorum, Desideria pumila, Oxyria digyna, Plantago euryphylla, Rhinathus minor, Rhododendron aureum, Salix herbacea, Saxifraga bryoides, Saxifraga nanella, Sedum alpestre, Silene ciliata, Solidago multiradiata, Veronica bellidioides, Waldheimia tridactylites*
1	237	*Achillea clusiana, Acinos alpinus, Aconitum spicatum, Adesmia erinacea, Adesmia spinosissima, Agrostis rupestris, Allionia incarnata, Aloysia deserticola, Alyssum klimesii, Ambrosia artemisioides, Androsace tapete, Anthyllis alpestris, Anthyllis vulneraria, Aphragmus oxycarpus, Arabis aculeolata, Arabis alpina, Arabis caerulea, Arenaria polytrichoides, Aristida adscensionis, Artemisia genipi, Artemisia tridentata, Atriplex imbricata, Azorella atacamensis, Azorella madreporica, Azorella monantha, Azorella selago, Baccharis boliviensis, Baccharis tola, Betula nana, Boechera lemmonii, Bouteloua gracilis, Bouteloua simplex, Brachyscome nivalis, Brachyscome scapigera, Calamagrostis cabrerae, Calamagrostis crispa, Calamagrostis purpurascens, Campanula pulla, Campanula raineri, Caragana gerardiana, Caragana versicolor, Carex humilis, Carex pallescens, Carex pilulifera, Carex sagaensi, Carex scabrirostris, Carex sempervirens, Cassiope tetragona, Celmisia haastii, Celmisia prorepens, Celmisia sericophylla, Celmisia viscosa, Centaurea montana, Cerastium alpinum, Cerastium arvense, Chorizanthe commissuralis, Chuquiraga atacamensis, Cirsium scopulorum, Clarkia xantiana* ssp. *xantiana, Claytonia perfoliata, Clinopodium alpinum, Coespeletia lutescens, Craspedia auratia, Dasiphora fruticose, Delphinium barbeyi, Delphinium nuttallianum, Deschampsia cespitosa, Dianthus sylvestris, Draba altaica, Draba oreades, Dracophyllum continentis, Dracophyllum muscoides, Dryas intergrifolia, Elymus nutans, Elymus scribneri, Ephedra americana, Erigeron clokeyi, Erigeron flagellaris, Erigeron multiradiatus, Erigeron speciosus, Erigeron uniflorus, Erythronium montanum, Erytrichium hemisphaericum, Exodeconus integrifolius, Fabiana denudata, Fagonia chilensis, Fargesia adpressa, Fargesia angustissima, Fargesia melanostachys, Festuca appenina* × *pratensis, Festuca brachyphylla, Festuca halleri agg., Festuca ovina* subsp. *Laevigata, fractional vegetation cover, Frasera speciosa, Gamocarpha ventosa, Gentiana bavarica, Gentiana lutea* subsp. *Lutea, Gentiana nipponica, Geum montanum, Geum reptans, Gnaphalium norvegicum, Gnaphalium supinum, Hebe odora, Helictochloa versicolor, Helictotrichon versicolor, Homogyne alpina, Horkelia sericata, Hydrophyllum capitatum, Hydrophyllum fendleri, Hypericum laricifolium, Hypericum maculatum, Jacobaea incana, Jarava frigida, Juncus falcatus, Juniperus communis, Kobresia humilis, Kobresia myosuroides, Koenigia islandica, Ladakiella klimesii, Laretia acaulis, Leucanthemopsis alpina, Leucochrysum alpinum, Linaria alpina, Lotus alpinus, Lupinus oreophilus, Lupinus subinflatus, Luzula novae‐cambriae, Luzula spicata, Mertensia ciliata, Mertensia fusiformis, Microgynoecium tibeticum, Microseris lanceolata, Minuartia gerardii, Minuartia sedoides, Moschopsis monocephala, Munroa decumbens, Myricaria elegans, Nabalus boottii, Nabalus trifoliolatus vsr. nanus, Nabalus trifoliolatus vsr. Nanus, Nardus stricta, Nassella nardioides, Noccaea corymbosa, Noccaea magellanica, Olearia frostii, Onobrychis montana, Oreochloa disticha, Pappochroma bellidioides, Pappochroma nitidum, Parastrephia quadrangularis, Pegaeophyton scapiflorum, Penstemon davidsonii, Penstemon newberryi, Phacelia corymbosa, Phacelia pinnatifida, Phacelia secunda, Phleum alpinum, Phyteuma hemisphaericum, Picrorhiza kurroa, Plant–soil mesocosms, Plantago lanceolata, Plantago major, Poa alpina var. vivipara, Poa hiemata, Poa laxa, Poa pratensis, Podolepis laciniata, Podolepis robusta, Polemonium viscosum, Polygonum cuspidatum, Polygonum weyrichii var. Alpinum, Potentilla anserine, Potentilla cuneata, Potentilla hippina* × *pulcherrima, Potentilla nicea, Potentilla parmirica, Potentilla pulcherrima, Prenanthes roanensis, Primula glutinosa, Primula sikkimensis, Pycnophyllum bryoides, Ranunculus alpestris, Rhododendron aganniphum, Rhododendron anthopogon, Rorippa elata, Rumex alpinus, Rytidosperma nudiflorum, Rytidosperma pictum, Sagina saginoides, Salix arctica, Salix flabellaris, Salix oritrepha, Salix pulchra, Salix richardsonii, Saussurea hypsipeta, Saussurea inversa, Saussurea nepalensis, Saxifraga cernua, Saxifraga cespitosa, Saxifraga exarata, Saxifraga moschata, Saxifraga oppositifolia, Saxifraga tolmiei, Scleranthus biflorus, Scorzoneroides helvetica, Senecio aethnensis, Senecio linearifolius, Senecio pinnatifolius, Senecio puchii, Sibbaldia procumbens, Silene vulgaris* ssp. *glareosa, Solanum chilense, Stellaria decumbens, Stellaria depressa, Stipa aliena, Succisa pratensis, Tagetes multiflora, Tanacetum alpinum, Taraxacum officinale, Thylacospermum caespitosum, Townsendia condensata, Trichocline caulescens, Trifolium alpinum, Trifolium pratense* ssp. *nivale, Tussilago farfara, Vahlodea atropurpurea, Veronica alpina, Veronica officinalis, Viola canina, Waldheimia stricta, Wittsteinia vacciniacea, Xerochrysum subundulatum, Youngia gracilipes, Yushania brevipaniculata, Yushania farinosa, Yushania weixiensis*

**TABLE A3:** Details on the nine most studied species.

Species name	Factor(s)	Limit(s)	Continent	Mountain range
*Poa alpina*	Plant–plant interactions (4), Temperature (2), Plant–animal interactions (1), Colonization (1), Topoclimatic (1)	Both	Europe, North America	Alps, Rocky Mountains
*Ranunculus glacialis*	Temperature (4), Colonization (1), Topoclimatic (1), Plant–plant interactions (1), Plant–animal interactions (1), Nutrients (1)	Upper	Europe	Alps
*Plantago alpina*	Plant–plant interactions (4), Selection and local adaptation (1), Water availability (1), Plant–animal interactions (1)	Lower	Europe	Alps
*Anthyllis vulneraria* ssp. *alpestris*	Plant–plant interactions (2), Selection and local adaptation (1), Plant animal interactions (1), Growing season (1)	Lower	Europe	Alps
*Boecheria stricta*	Selection and local adaptation (3), Water availability (2), Plant–animal interactions (2)	Both	North America	Rocky Mountains, Great Plains
*Poa attenuata*	Temperature (2), Growing season (1), Soil (1), Water availability (1)	Upper	Asia	Karakoram
*Sesleria caerulea*	Plant–plant interactions (3), Plant–animal interactions (1)	Lower	Europe	Alps
*Silene acaulis*	Temperature (2), Topoclimatic (1), Plant–plant interactions (1), Colonization (1)	Both	Europe	Alps
*Trifolium badium*	Plant–plant interactions (4), Water availability (1)	Lower	Europe	Alps

*Note:* Only species present in more than two studies are reported. The numbers behind the factors indicate how often the factor was studied on this species.

## Appendix C

### Global Distribution of Observations

The vast majority of observations (82%) were conducted in the Northern Hemisphere, with limited research effort in the tropics (1%), the boreal zone (4%), and the Arctic (2%; Figure A1), highlighting a strong geographic bias in the scientific literature.
